# Baricitinib in the Management of Severe Alopecia Areata: A Report of Two Cases With Sustained Clinical Response

**DOI:** 10.7759/cureus.94278

**Published:** 2025-10-10

**Authors:** Zemmez Youssef, Mohamed-amine Ennaciri, Rachid Frikh, Naoufal Hjira

**Affiliations:** 1 Dermatology Department, Mohamed V Military Hospital, Rabat, MAR

**Keywords:** alopecia areata, baricitinib, hair regrowth, janus kinase inhibitors, salt score

## Abstract

Baricitinib has recently received approval from the US Food and Drug Administration (FDA) as a promising treatment option for adults with severe alopecia areata. Beyond this indication, it has demonstrated efficacy in other conditions such as COVID-19 and rheumatoid arthritis. The drug also presents advantages related to cost, minimal invasiveness, and the absence of postoperative management. Nevertheless, its prescription requires careful consideration of potential safety and clinical implications before widespread use. We describe two patients with severe alopecia areata (baseline Severity of Alopecia Tool (SALT) score = 100), who were treated with baricitinib as an alternative to conventional therapies, both of whom showed favorable outcomes with a progressive reduction in SALT score leading to complete regrowth during a 12-month follow-up period.

## Introduction

Alopecia areata (AA) is a common, chronic, immune-mediated disorder that affects the hair follicle and results in non-scarring hair loss. Its prevalence is estimated at 0.1-0.2% worldwide, with a lifetime risk of approximately 2% in the general population [1]. Clinically, AA presents as well-defined patches of hair loss on the scalp or body. In more severe forms, the disease may progress to alopecia totalis (complete scalp hair loss) or alopecia universalis (loss of all scalp and body hair). The term “non-scarring” indicates that the follicular structures remain intact, thus allowing the potential for regrowth, in contrast with scarring alopecia, where follicles are permanently destroyed. The disease can manifest at any age, frequently in childhood or adolescence, and has an unpredictable course characterized by relapses and remissions. While AA is not life-threatening, its impact on patients’ quality of life is profound, contributing to emotional distress, low self-esteem, depression, and social anxiety [2,3].

The pathogenesis of AA involves a complex interplay between genetic susceptibility, environmental triggers, and immune dysregulation. Under physiological conditions, hair follicles maintain an “immune privilege,” protecting them from autoimmune attack. In AA, this privilege is disrupted, leading to infiltration by autoreactive CD8+ T lymphocytes activated by cytokines such as interferon-γ (IFN-γ) and interleukin-15 (IL-15). These signals are transmitted through the Janus kinase (JAK)-STAT pathway, providing a therapeutic rationale for JAK inhibition [4,5].

Conventional treatments, including topical or systemic corticosteroids, intralesional corticosteroid injections, immunosuppressants such as methotrexate, and topical minoxidil, often yield incomplete or transient responses, particularly in severe forms of AA. Treatment goals generally include promoting hair regrowth and preventing further disease progression; however, patients with extensive or refractory disease remain a major therapeutic challenge [6].

Recent clinical advances have demonstrated that JAK inhibitors, such as tofacitinib, ruxolitinib, and, more recently, baricitinib, can promote substantial hair regrowth in patients with severe AA [4,7]. On June 13, 2022, the US Food and Drug Administration (FDA) approved oral baricitinib as the first systemic therapy for adult patients with severe AA, marking a major milestone in the management of this disease [8]. Compared with other JAK inhibitors, baricitinib is currently the only one with regulatory approval for AA and has shown robust efficacy in phase 3 trials. Severe AA in clinical practice is generally defined by extensive scalp involvement, often quantified by a Severity of Alopecia Tool (SALT) score ≥50, which allows objective monitoring of disease burden and response to therapy. Moreover, clinical improvement with JAK inhibitors is usually observed within three to four months, with complete regrowth achievable in some cases after nine to 12 months of continuous therapy [9,10].

Despite these advances, real-world data remain relatively limited compared with clinical trials, and documenting outcomes in routine practice is essential to better understand efficacy, safety, and patient tolerance.

In this article, we report two cases of severe, treatment-resistant AA managed with baricitinib. These cases highlight the clinical utility, sustained efficacy, and favorable tolerability of this therapy, providing valuable complementary evidence to the existing literature.

## Case presentation

Case 1

**Figure 1:**
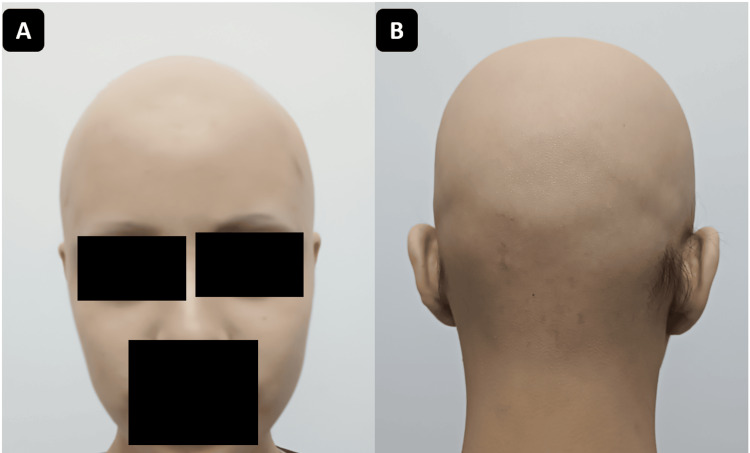
Case 1: Complete hair loss consistent with severe alopecia areata. The Severity of Alopecia Tool (SALT) score is estimated at 100. A: Anterior view. B: Posterior view.

An 18-year-old female patient, with no relevant medical history, presented with progressive hair loss that initially manifested as two alopecic patches on the vertex and extended over six months to involve the entire scalp and partially the eyebrows. On admission, dermatological examination revealed complete scalp alopecia and approximately 20% eyebrow involvement, without loss of eyelashes or nail changes (Figure 1).

**Figure 2:**
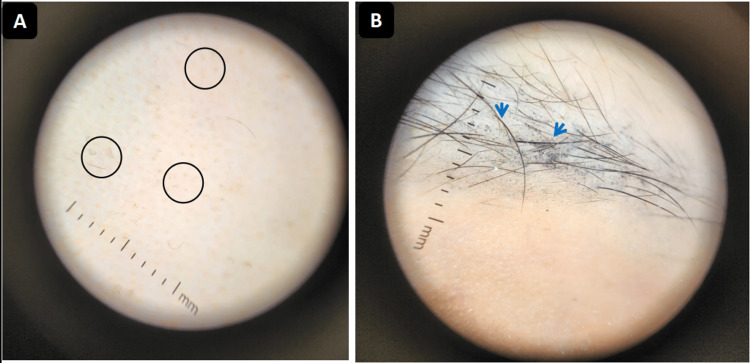
Case 1: Dermoscopic examination showing features of alopecia areata. A: Scalp dermoscopy demonstrating black dots, yellow dots, and bent hairs (circle). B: Eyebrow dermoscopy showing broken and sparse hairs (arrow).

Trichoscopy demonstrated black dots (destroyed hairs within follicles), yellow dots (keratin-filled follicular openings), broken hairs (signs of follicular damage), and bent or fine vellus hairs (indicating early regrowth) on the scalp (Figure 2A) and on the eyebrows (Figure 2B). These findings were consistent with alopecia areata. The SALT score was estimated at 100.

The disease followed a stepwise course: initial localized alopecic patches were noted in the first month after symptom onset, which progressively extended to complete scalp involvement by the sixth month. The patient was initially treated with topical corticosteroids and monthly intralesional triamcinolone injections for three months without improvement, followed by methotrexate (15 mg/week) combined with oral mini-pulse (“weekend”) corticosteroid therapy consisting of dexamethasone ~0.1 mg/kg/day administered on two consecutive days each week for six months, with clinical and laboratory monitoring. Despite these interventions, there was no improvement. She was subsequently hospitalized on three occasions for intravenous methylprednisolone pulses (15 mg/kg/day for three consecutive days), with unsatisfactory results.

After careful review of personal and family history, no autoimmune comorbidities, infections, psychological stressors, or environmental triggers were identified as potential precipitating factors.

Given the refractory course, baricitinib (4 mg/day) was initiated six weeks after the last corticosteroid pulse to ensure adequate washout of prior therapies. Before initiation, a comprehensive baseline evaluation was performed, including interferon gamma release assay (IGRA, i.e., Quantiferon), C-reactive protein (CRP), liver and renal function tests, creatine phosphokinase (CPK), lipid profile, and viral serologies for hepatitis B virus (HBV), hepatitis C virus (HCV), and human immunodeficiency virus (HIV). Baseline laboratory values were within normal limits (alanine aminotransferase (ALT) = 22 U/L, aspartate aminotransferase (AST) = 24 U/L, creatinine = 0.8 mg/dL, total cholesterol = 175 mg/dL, CPK = 110 U/L, CRP = 3 mg/L). Follow-up monitoring at three, six, nine, and 12 months showed stable results with no abnormalities detected.

**Figure 3:**
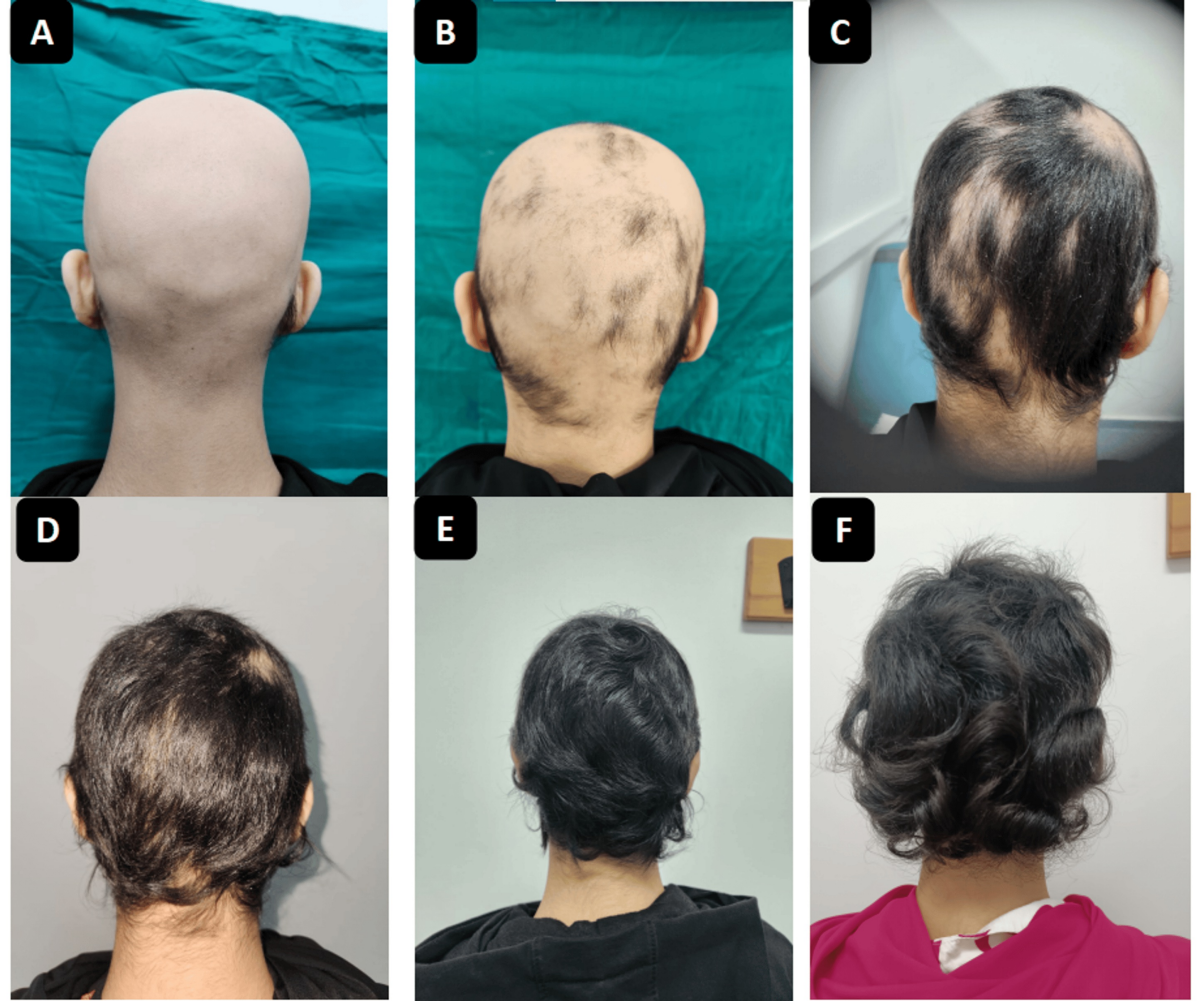
Case 1: Favorable clinical evolution under treatment. A: Appearance after one month of treatment; SALT score = 93. B: Appearance after two months of treatment; SALT score = 62. C: Appearance after three months of treatment; SALT score = 34. D: Appearance after six months of treatment, SALT score = 9. E: Appearance after nine months of treatment; SALT score = 0. F: Appearance after 12 months of treatment; SALT score = 0. SALT: Severity of Alopecia Tool.

Clinical follow-up was scheduled monthly for the first three months (month one: M1 (Figure 3A), month two: M2 (Figure 3B), month three: M3 (Figure 3C)), and then quarterly (M6 (Figure 3D), M9 (Figure 3E), M12 (Figure 3F)), with regular laboratory monitoring.

**Figure 4:**
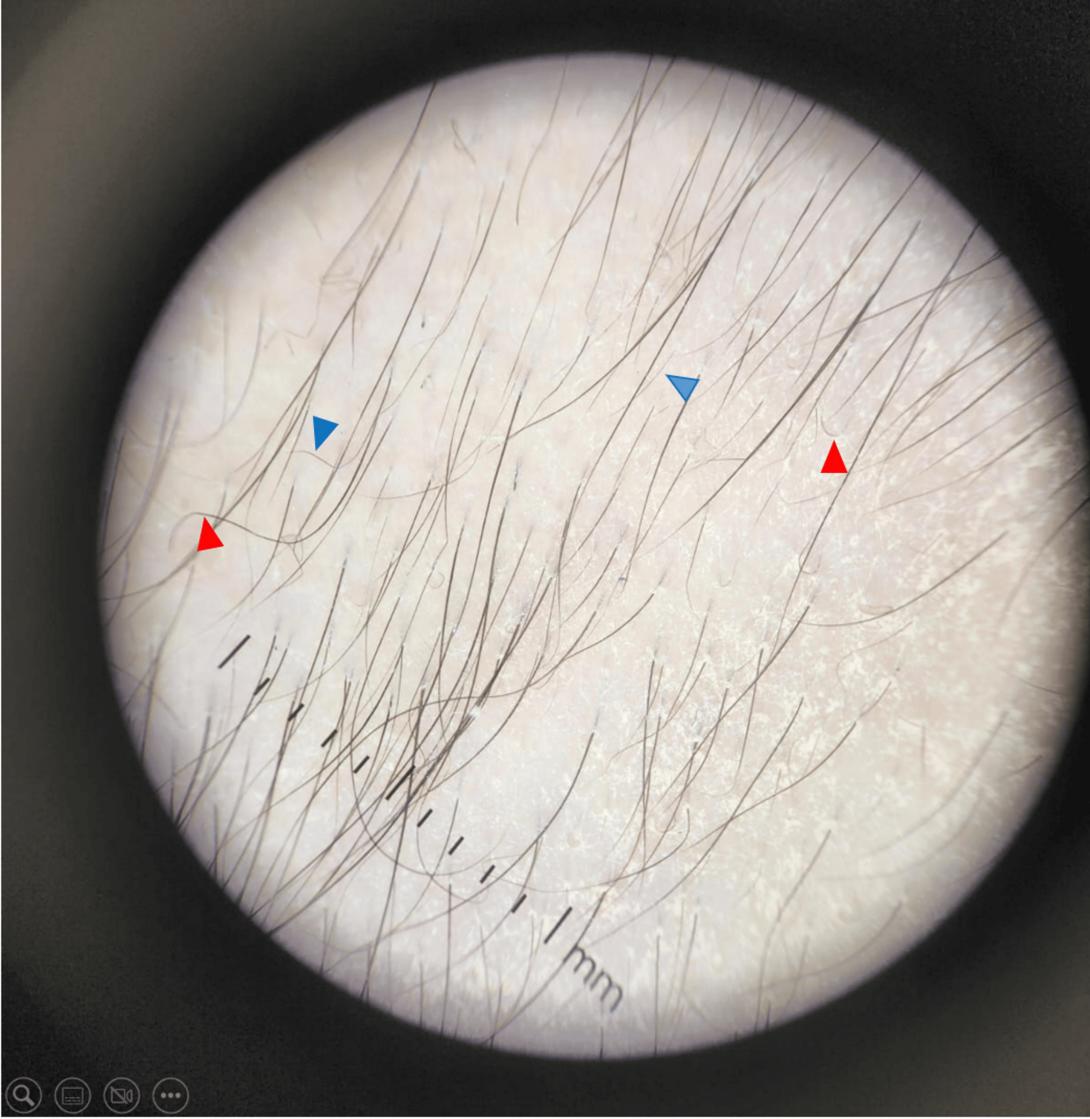
Case 1: Scalp dermoscopy after three months of treatment showing signs of regrowth: vellus hairs (blue arrows) and coiled hairs (red arrows).

Clinical evolution was favorable, with progressive regrowth on the scalp and eyebrows. Dermoscopy confirmed regrowth with coiled hairs, straight regrowing hairs, and vellus hairs (Figure 4).

SALT scores improved markedly (93 at month one, 62 at month two, 34 at month three, 9 at month six, and 0 at month nine), corresponding to complete regrowth. The patient achieved a 50% reduction in SALT score (SALT =50) between months two and three, indicating a rapid response rate.

Treatment was well tolerated. The patient reported intermittent headaches (two to three episodes per month, mild intensity, resolving spontaneously without medication) and mild dandruff. No laboratory or systemic abnormalities occurred throughout the 12-month course.

Case 2

**Figure 5:**
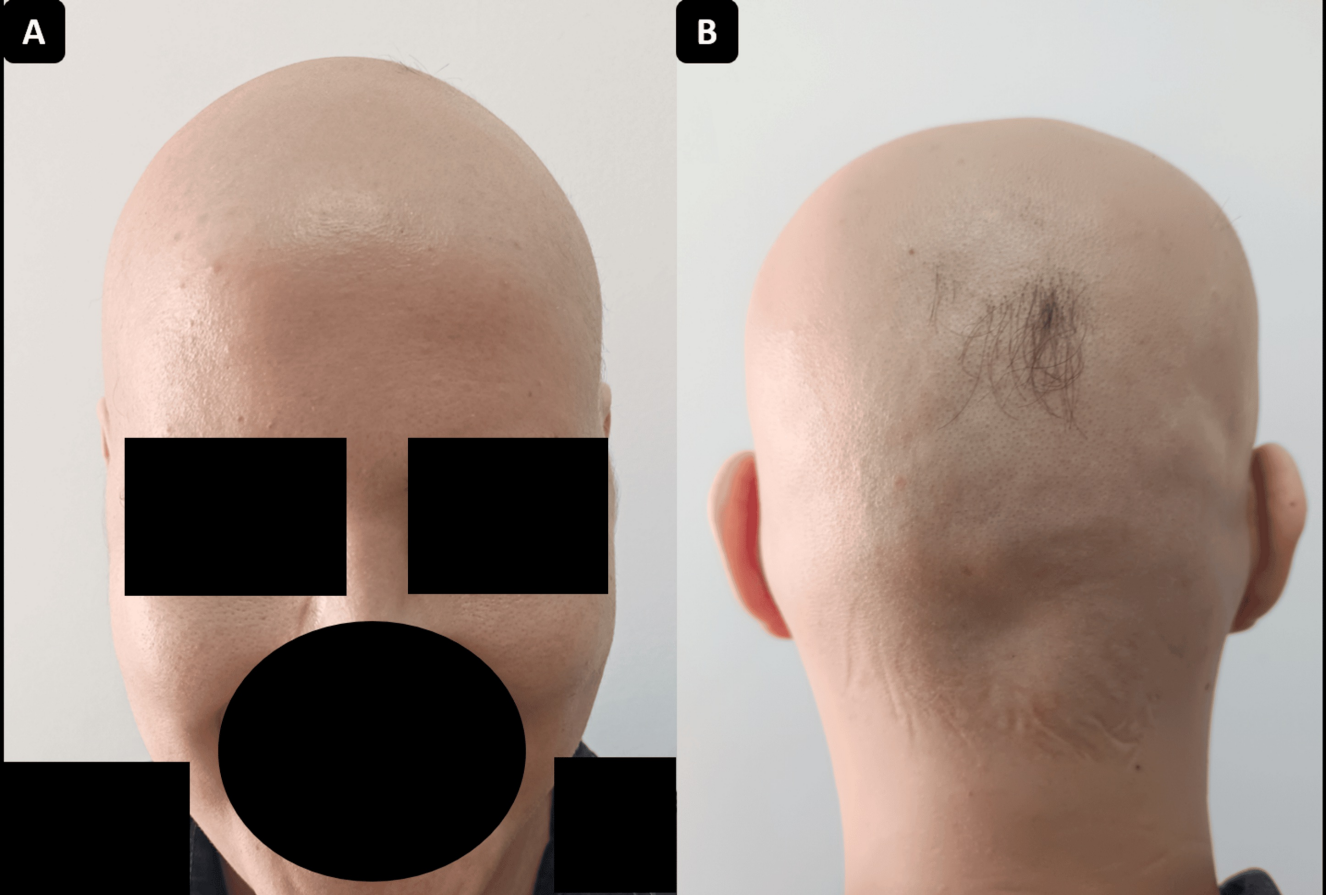
Case 2: Complete hair loss. The Severity of Alopecia Tool (SALT) score is estimated at 100. A: Anterior view. B: Posterior view.

A 30-year-old male patient, with no relevant medical history, presented with progressive hair loss that began as several diffuse alopecic patches and progressed over four months to involve the entire scalp. Dermatological examination revealed complete scalp alopecia, with no eyebrow, eyelash, or nail involvement (Figure 5). Dermoscopy demonstrated yellow dots (keratin-filled follicular openings), black dots (destroyed hairs within follicles), broken hairs (active follicular damage), angled hairs, and fine vellus hairs (early regrowth markers), consistent with alopecia areata. The SALT score was 100.

The progression timeline was rapid: multiple alopecic patches appeared within the first month after symptom onset, coalesced by the third month, and resulted in complete scalp alopecia by the fourth month. Initial therapies, including methotrexate (15 mg/week) combined with intralesional triamcinolone and topical minoxidil for six months, were ineffective in halting disease progression. No triggering factors were identified after reviewing the patient’s history, including infections, systemic illnesses, or psychosocial stress.

Baricitinib was introduced four weeks after discontinuing methotrexate to ensure an adequate washout period. Baricitinib (4 mg/day) was initiated following a complete baseline evaluation, which excluded contraindications. Baseline laboratory tests showed normal results (ALT = 25 U/L, AST = 26 U/L, creatinine = 0.9 mg/dL, total cholesterol = 180 mg/dL, CPK = 105 U/L, CRP - 2 mg/L). At month three, total cholesterol rose mildly to 220 mg/dL and stabilized at 215 mg/dL thereafter, while all other parameters remained normal.

**Figure 6:**
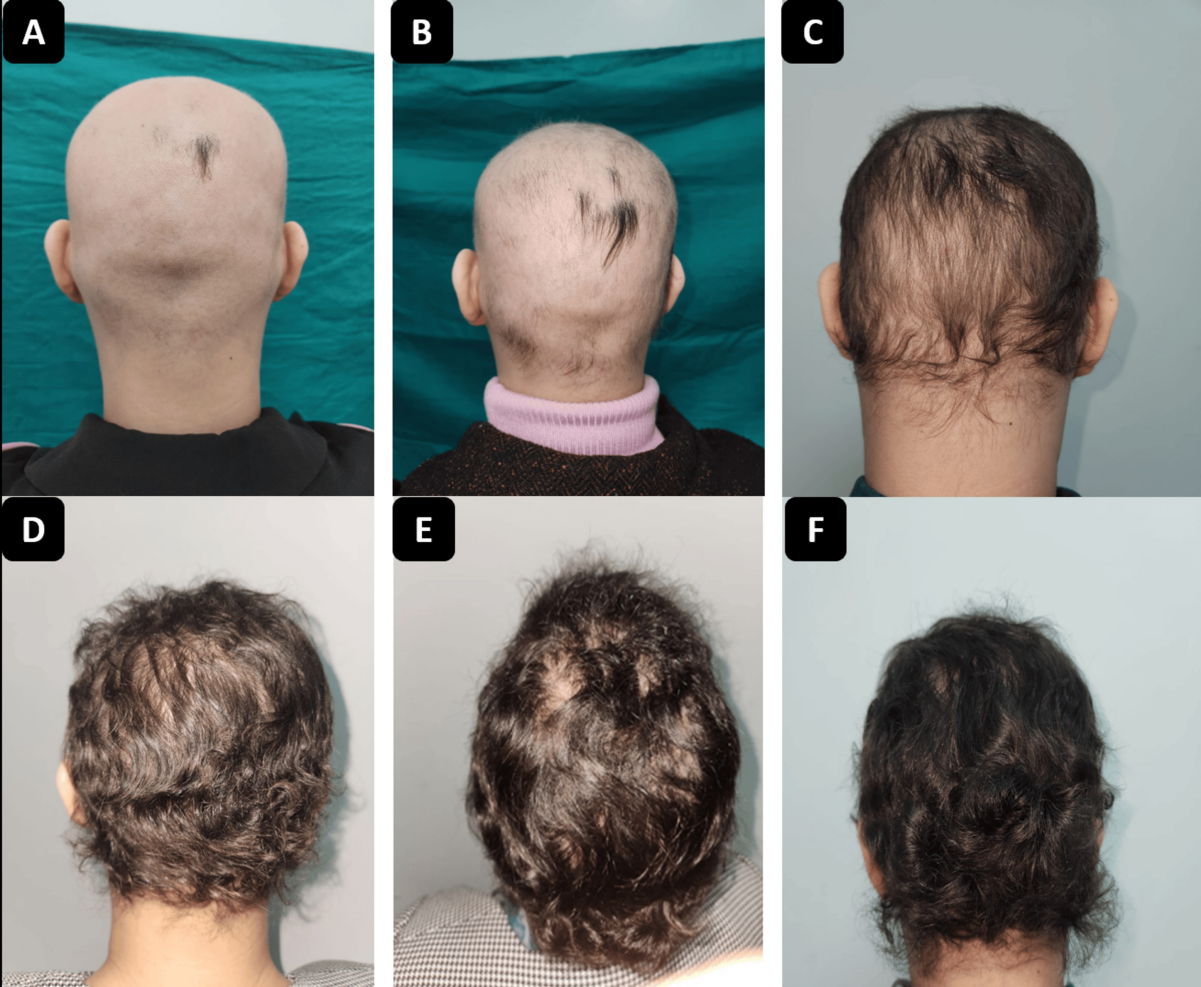
Case 2: Favorable clinical evolution under treatment. A: Appearance after one month of treatment; SALT score = 95. B: Appearance after two months of treatment; SALT score = 60. C: Appearance after three months of treatment; SALT score = 23. D: Appearance after six months of treatment; SALT score = 5. E: Appearance after nine months of treatment; SALT score = 3. F: Appearance after 12 months of treatment; SALT score = 0. SALT: Severity of Alopecia Tool.

Monitoring was scheduled monthly for the first three months and then quarterly, with laboratory assessments. The clinical course was favorable, with progressive scalp hair regrowth confirmed by dermoscopy showing coiled hairs, straight regrowing hairs, and vellus hairs. Significant improvement in SALT scores was observed during follow-up (Figure 6), with assessments at one month (Figure 6A), two months (Figure 6B), three months (Figure 6C), six months (Figure 6D), nine months (Figure 6E), and 12 months (Figure 6F). The patient achieved a 50% reduction in SALT score (SALT = 50) between months two and three and complete regrowth (SALT = 0) by month 12.

Treatment was well tolerated throughout the 12-month course of baricitinib therapy. The only adverse event observed was a mild, transient increase in total cholesterol (from 180 mg/dL at baseline to 220 mg/dL at month three, then 215 mg/dL at month 12), which did not require any therapeutic adjustment. Minimal dandruff was also noted but required no intervention.

Table 1 provides a comparative overview of both patients, including baseline characteristics, prior treatments, laboratory findings, SALT score evolution, and clinical outcomes under baricitinib therapy.

**Table 1:** Comparative summary of the two cases treated with baricitinib. SALT: Severity of Alopecia Tool; ALT: alanine aminotransferase; AST: aspartate aminotransferase; CRP: C-reactive protein; IGRA: interferon gamma release assay; CPK: creatine phosphokinase.

Parameter	Case 1	Case 2
Age/sex	18 years, female	30 years, female
Disease duration before baricitinib	6 months (patchy → total scalp alopecia)	4 months (diffuse patches → total scalp alopecia)
Baseline clinical findings	Complete scalp alopecia, ~20% eyebrow loss, no eyelash/nail involvement	Complete scalp alopecia, no eyebrow/eyelash/nail involvement
Washout period before baricitinib	6 weeks after the last corticosteroid pulse	4 weeks after discontinuing methotrexate
Baricitinib regimen	4 mg/day	4 mg/day
Baseline laboratory values	Hepatic panel: ALT = 22 U/L, AST = 24 U/L. Renal panel: creatinine = 0.8 mg/dL. Lipid panel: total cholesterol = 175 mg/dL. CRP = 3 mg/L. Quantiferon (IGRA): Negative. CPK = 110 U/L.	Hepatic panel: ALT = 25 U/L, AST = 26 U/L. Renal panel: creatinine = 0.9 mg/dL. Lipid panel: total cholesterol = 180 mg/dL. CRP = 2 mg/L. Quantiferon (IGRA): Negative. CPK = 105 U/L.
Follow-up laboratory results	Stable and within normal range at all time points	Mild increase in cholesterol (220 mg/dL at month 3, 215 mg/dL at month 12); others stable
Adverse events	Intermittent mild headaches (2–3/month), mild dandruff	Mild transient hypercholesterolemia, minimal dandruff
Baseline SALT score	100	100
Time to 50% improvement (SALT = 50)	Between months 2 and 3	Between months 2 and 3
Time to 100% improvement (SALT = 0)	9 months (complete regrowth)	12 months (complete regrowth)

## Discussion

Alopecia areata (AA) is a complex autoimmune condition with heterogeneous clinical outcomes. Recent advances have identified Janus kinase inhibitors (JAKi) as an effective therapeutic option for severe or refractory cases. Among them, baricitinib, a selective JAK1/JAK2 inhibitor, has shown consistent efficacy in clinical trials and is currently the only FDA-approved systemic therapy for severe AA [4]. Our report contributes real-world evidence of baricitinib use, with specific clinical characteristics that distinguish our observations from prior reports.

Both of our patients achieved significant and sustained regrowth with baricitinib, reaching a SALT score <50 after three months and complete regrowth (SALT = 0) at nine months. These results are in line with the BRAVE-AA1 and BRAVE-AA2 phase 3 trials, in which approximately 35-40% of patients treated with baricitinib 4 mg achieved ≥80% scalp coverage by week 36 [7,8]. Case series and observational studies have reported variable responses, often partial or requiring longer treatment durations. The rapid and complete regrowth in our patients underscores the potential for robust responses in selected cases, even after multiple treatment failures [11].

The early clinical improvement observed within three months is consistent with data from pivotal trials, where initial regrowth was typically noted between weeks 12 and 16. However, the achievement of complete regrowth by nine months in both patients may represent a particularly favorable outcome compared with averages reported in larger cohorts, where some patients required one year or more for maximal response [7,11].

**Figure 7:**
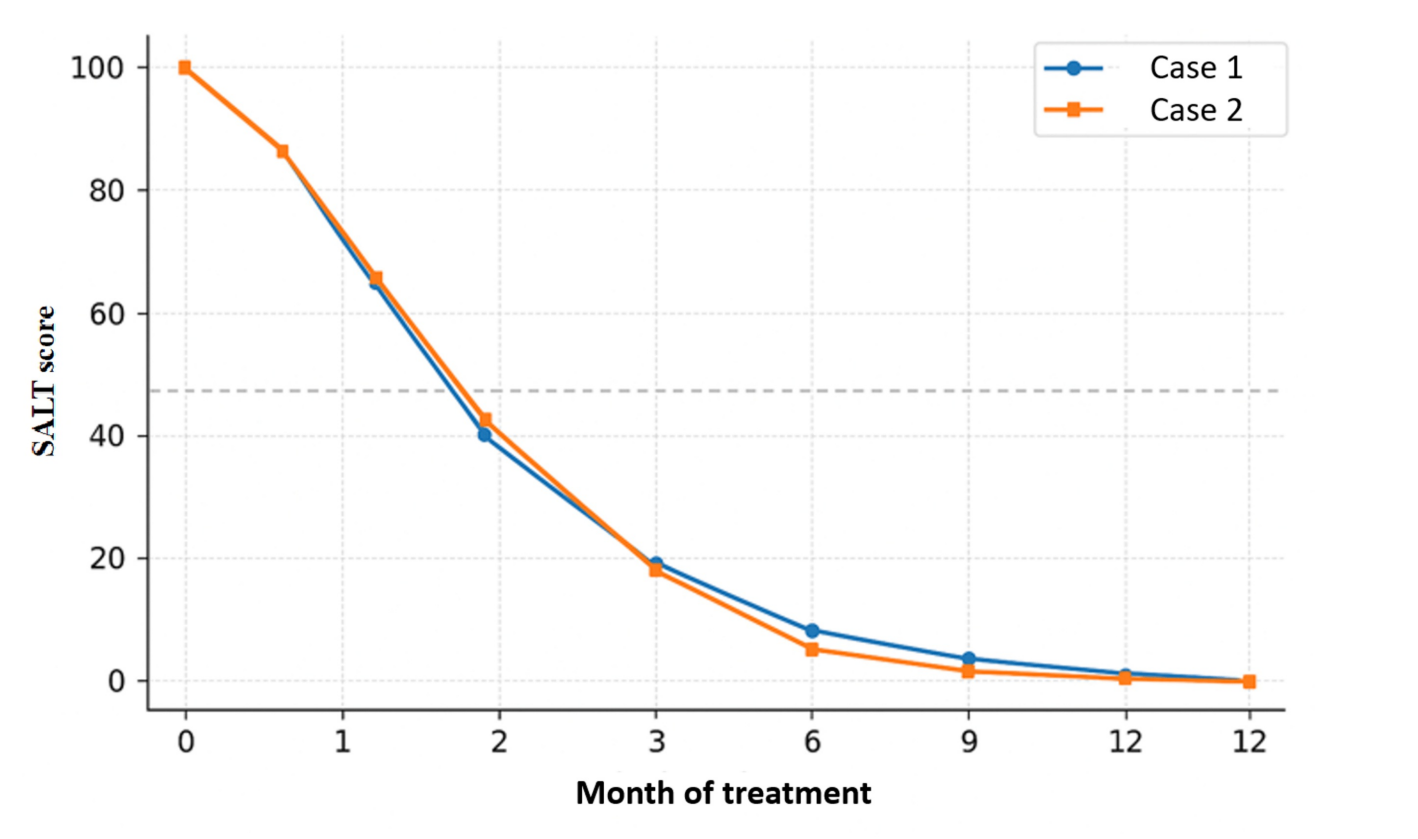
Comparative evolution of the Severity of Alopecia Tool (SALT) score under baricitinib therapy for the cases presented in this report.

An important strength of our report is the systematic use of the SALT score at each follow-up visit, as illustrated in Figure 7, where both patients demonstrated a progressive and sustained reduction in disease severity, reaching complete remission by month 12. While many published case reports describe qualitative improvement, few provide quantitative scoring. This objective measure not only confirms the efficacy of treatment but also facilitates comparison with clinical trial data.

In addition, dermoscopy was performed systematically and provided insight into the regrowth process. Features such as coiled hairs, regrowing straight hairs, and vellus hairs were documented as early markers of follicular reactivation. Previous reports rarely integrate dermoscopy into longitudinal follow-up, highlighting the novelty of our approach [12].

Treatment was well tolerated in both patients. Only mild adverse effects were observed: intermittent headaches and dandruff in the first case, and a modest increase in total cholesterol in the second. These findings are reassuring and consistent with safety data from clinical trials, where infections, laboratory abnormalities, and thromboembolic risks were the main concerns [7,13]. The absence of significant laboratory changes in our patients adds to the accumulating real-world evidence of acceptable tolerability, although vigilance remains essential [13].

Our patients were followed for 12 months, providing insight into the durability of response. While some case reports describe shorter follow-up periods, our report demonstrates sustained remission under continuous therapy, with no evidence of relapse during the observation period [11,12]. This prolonged follow-up contributes meaningful data to the limited real-world literature.

The optimal duration of baricitinib therapy in AA remains uncertain [11,12]. In our experience, continuous treatment for 12 months was required to achieve and maintain complete regrowth. Published data suggest that relapse can occur in a subset of patients after discontinuation [9], emphasizing the need for individualized treatment duration and gradual tapering when possible. Long-term studies beyond one year are needed to define maintenance strategies and relapse predictors [12].

From a pharmacoeconomic perspective, baricitinib is generally less costly than newer selective JAK inhibitors, such as ritlecitinib or deuruxolitinib, particularly in regions where generic formulations or hospital procurement systems reduce price differentials [14]. Nevertheless, cost remains a barrier to widespread use, and future health-economic analyses comparing JAK inhibitors would be of great value.

The more favorable response observed in our two patients compared with phase 3 trial averages (BRAVE-AA1/2) may reflect multiple factors: earlier treatment initiation after disease stabilization, strict adherence, and the absence of major comorbidities [10]. Genetic or immunologic variability, including cytokine or JAK-STAT signaling differences, may also contribute to interindividual response heterogeneity [12].

From a clinical perspective, our findings reinforce the role of baricitinib as a therapeutic option for adults with severe or refractory AA who have not responded to corticosteroids or immunosuppressants. Ideal candidates are those with extensive scalp involvement (SALT > 50), absence of contraindications to JAK inhibition, and willingness to undergo baseline and periodic laboratory monitoring (including blood counts, liver function, and lipid profile). Baricitinib should be considered only after a comprehensive risk-benefit evaluation and shared decision-making with the patient. The favorable outcomes in our cases suggest that early initiation in active disease and continuous adherence may enhance efficacy. Nevertheless, careful long-term monitoring remains essential given the potential for relapse after discontinuation and the limited data beyond one year of therapy [9,10].

Finally, this report has several inherent limitations related to its case-based design. It includes only two patients and lacks a control group, which limits statistical interpretation and generalizability. In addition, as both patients were recruited from a tertiary referral center and had severe, treatment-refractory disease, a selection bias toward more motivated or clinically favorable profiles cannot be excluded. The absence of randomization or blinding also precludes control over potential confounding variables. Nevertheless, the systematic and quantitative documentation, including regular SALT scoring, dermoscopic monitoring, and longitudinal laboratory follow-up, strengthens the internal validity of these observations and provides meaningful real-world insight into baricitinib’s sustained efficacy and safety in severe AA.

In summary, our cases confirm the efficacy and safety of baricitinib for severe, treatment-resistant AA, with rapid and complete regrowth, systematic monitoring through SALT scoring and dermoscopy, and sustained response over one year. Compared with prior reports, the originality of our contribution lies in the detailed quantitative evaluation, integration of trichoscopic findings, and documentation of long-term tolerance in patients refractory to multiple conventional therapies.

## Conclusions

In summary, our cases illustrate the efficacy and safety of baricitinib in two patients with severe, treatment-resistant alopecia areata, who achieved rapid and sustained hair regrowth under continuous therapy. Systematic monitoring through SALT scoring and dermoscopy provided objective confirmation of progressive improvement and durable response over one year, with no significant adverse events. Our report adds original clinical insights by combining long-term follow-up, quantitative scoring, trichoscopic documentation of regrowth, and detailed safety monitoring. While these findings align with previously published data, they should be interpreted with caution, given the small sample size and case-based nature of this report. Larger prospective studies and long-term real-world data are needed to further validate these observations and optimize therapeutic strategies for severe alopecia areata.

## References

[1] Sibbald C (2023). Alopecia areata: an updated review for 2023. J Cutan Med Surg.

[2] Liu LY, King BA, Craiglow BG (2018). Alopecia areata is associated with impaired health-related quality of life: a survey of affected adults and children and their families. J Am Acad Dermatol.

[3] Okhovat JP, Marks DH, Manatis-Lornell A, Hagigeorges D, Locascio JJ, Senna MM (2023). Association between alopecia areata, anxiety, and depression: a systematic review and meta-analysis. J Am Acad Dermatol.

[4] Lin X, Li X, Zhai Z, Zhang M (2025). JAK-STAT pathway, type I/II cytokines, and new potential therapeutic strategy for autoimmune bullous diseases: update on pemphigus vulgaris and bullous pemphigoid. Front Immunol.

[5] Miot HA, Criado PR, de Castro CCS, Ianhez M, Talhari C, Ramos PM (2023). JAK-STAT pathway inhibitors in dermatology. An Bras Dermatol.

[6] Juárez-Rendón KJ, Rivera Sánchez G, Reyes-López MÁ, García-Ortiz JE, Bocanegra-García V, Guardiola-Avila I, Altamirano-García ML (2017). Alopecia areata. Current situation and perspectives. Arch Argent Pediatr.

[7] Zhao HB, Zhang YN, Qiang Y, Wang GM, Wang LW, Jiang WC, Chen X (2025). From mechanisms to therapies: current advances breakthroughs in alopecia areata immunopathology. Front Immunol.

[8] (2025). FDA approves first systemic treatment for alopecia areata. https://www.prnewswire.com/news-releases/fda-approves-first-systemic-treatment-for-alopecia-areata-301566884.html.

[9] De Greef A, Thirion R, Ghislain PD, Baeck M (2023). Real-life effectiveness and tolerance of baricitinib for the treatment of severe alopecia areata with 1-year follow-up data. Dermatol Ther (Heidelb).

[10] King B, Ohyama M, Kwon O (2022). Two phase 3 trials of baricitinib for alopecia areata. N Engl J Med.

[11] Vignoli CA, Gargiulo L, Ibba L (2025). Baricitinib for the treatment of severe alopecia areata: results from a 52-week multicenter retrospective real-world study. J Dermatolog Treat.

[12] Wada-Irimada M, Takahashi T, Sekine M (2025). Long-term real-world outcomes of baricitinib in severe alopecia areata: a 104-week retrospective analysis from a single Institute. J Dermatol.

[13] Saceda-Corralo D, Vañó-Galván S (2025). Strong efficacy of ritlecitinib 50 mg and baricitinib 4 mg in alopecia areata, but further research needed to establish superiority. J Eur Acad Dermatol Venereol.

[14] Malaniuk K (2025). Modern and promising strategies for treatment of alopecia in the United States: clinical and pharmacological, marketing, regulatory, SWOT analysis. SSP Mod Pharm Med.

